# Efficacy of a Non-Hypercalcemic Vitamin-D2 Derived Anti-Cancer Agent (MT19c) and Inhibition of Fatty Acid Synthesis in an Ovarian Cancer Xenograft Model

**DOI:** 10.1371/journal.pone.0034443

**Published:** 2012-04-03

**Authors:** Richard G. Moore, Thilo S. Lange, Katina Robinson, Kyu K. Kim, Alper Uzun, Timothy C. Horan, Nada Kawar, Naohiro Yano, Sharon R. Chu, Quanfu Mao, Laurent Brard, Monique E. DePaepe, James F. Padbury, Leggy A. Arnold, Alexander Brodsky, Tun-Li Shen, Rakesh K. Singh

**Affiliations:** 1 Molecular Therapeutics Laboratory, Program in Women's Oncology, Department of Obstetrics and Gynecology, Women and Infants' Hospital of Rhode Island, Alpert Medical School, Brown University, Providence, Rhode Island, United States of America; 2 Center for Computational Molecular Biology, Department of Pediatrics, Women and Infants' Hospital of Rhode Island, Alpert Medical School, Brown University, Providence, Rhode Island, United States of America; 3 Department of Pediatrics, Women and Infants' Hospital of Rhode Island, Alpert Medical School, Brown University, Providence, Rhode Island, United States of America; 4 Developmental Pathology, Women and Infants' Hospital of Rhode Island, Alpert Medical School, Brown University, Providence, Rhode Island, United States of America; 5 Gynecology Oncology, Southern Illinois Medical School, Springfield, Illinois, United States of America; 6 Chemistry and Biochemistry, University of Wisconsin-Milwaukee, Milwaukee, Wisconsin, United States of America; 7 Department of Biology and Medicine, Brown University, Providence, Rhode Island, United States of America; 8 Department of Chemistry, Brown University, Providence, Rhode Island, United States of America; Ludwig-Maximilians University, Germany

## Abstract

**Background:**

Numerous vitamin-D analogs exhibited poor response rates, high systemic toxicities and hypercalcemia in human trials to treat cancer. We identified the first non-hypercalcemic anti-cancer vitamin D analog MT19c by altering the A-ring of ergocalciferol. This study describes the therapeutic efficacy and mechanism of action of MT19c in both *in vitro* and *in vivo* models.

**Methodology/Principal Finding:**

Antitumor efficacy of MT19c was evaluated in ovarian cancer cell (SKOV-3) xenografts in nude mice and a syngenic rat ovarian cancer model. Serum calcium levels of MT19c or calcitriol treated animals were measured. In-silico molecular docking simulation and a cell based VDR reporter assay revealed MT19c–VDR interaction. Genomewide mRNA analysis of MT19c treated tumors identified drug targets which were verified by immunoblotting and microscopy. Quantification of cellular malonyl CoA was carried out by HPLC-MS. A binding study with PPAR-Y receptor was performed. MT19c reduced ovarian cancer growth in xenograft and syngeneic animal models without causing hypercalcemia or acute toxicity. MT19c is a weak vitamin-D receptor (VDR) antagonist that disrupted the interaction between VDR and coactivator SRC2-3. Genome-wide mRNA analysis and western blot and microscopy of MT19c treated xenograft tumors showed inhibition of fatty acid synthase (FASN) activity. MT19c reduced cellular levels of malonyl CoA in SKOV-3 cells and inhibited EGFR/phosphoinositol-3kinase (PI-3K) activity independently of PPAR-gamma protein.

**Significance:**

Antitumor effects of non-hypercalcemic agent MT19c provide a new approach to the design of vitamin-D based anticancer molecules and a rationale for developing MT19c as a therapeutic agent for malignant ovarian tumors by targeting oncogenic *de novo* lipogenesis.

## Introduction

Epithelial ovarian cancer (EOC) is the leading cause of death from gynecologic malignancies. Early-stage cancers are mostly asymptomatic, and most of the diagnoses at presentation detect established regional or distant metastases [1]. The majority of the patients will experience recurrent disease, as well as resistance to chemotherapeutic agents. The low survival rate of advanced stage ovarian cancer has made early detection, understanding the etiology of the disease and the targeting of specific characteristic features, as the top priorities in cancer research [1].

Increased *de novo* fatty-acid synthesis is a hallmark of cancer [2], [3]. Otto Warburg first observed enhanced anaerobic glycolysis in cancer cells [4]. Normal human tissues use dietary fats for synthesis of new structural lipids, whereas incessantly proliferating cancer cells for unknown reasons avoid utilization of dietary fats and carry out independent *de novo* fatty acid synthesis to continually provide for membrane production, energy generation and lipid modification of proteins [4]. *De novo* fatty-acid synthesis involves two key enzymes; acetyl Co-A carboxylase (ACC) and fatty-acid synthase (FASN). ACC carboxylates acetyl-CoA to form malonyl CoA. The malonyl-CoA product is further converted by FASN to long-chain fatty acids. Newly synthesized fatty acids are stored by lipolytic PPAR-gamma to avoid fatty acid toxicity. Therefore, deregulated functions of lipogenic enzymes FASN and ACC involved in *de novo* fatty-acid synthesis in conjunction with lipolytic PPAR-gamma play an important role in promoting tumor cell survival at multiple levels.

Numerous studies have shown overexpression of FASN in human epithelial ovarian cancer (EOC) [5]–[8] and cancers of breast [9], prostate [10], colon [11], lung [12], endometrium [13] and papillary thyroid [14]. An oligonucleotide microarrays consisting of more than 6,000 human genes identified fatty acid synthase (FASN) as a potential therapeutic or molecular target in EOC [15]. Subsequently, a pharmacological targeting of FASN by the natural product cerulenin and a synthetic molecule C75 suppressed growth of ovarian and breast cancer in animal models [16]–[17]

In the current study, we show that MT19c is a new class of antitumor agent that targets critical components of *de novo* fatty acid synthesis machinery in ovarian cancer xenograft tumors and ovarian cancer cells. MT19c is a novel vitamin-D2 derived agent that exerted no hypercalcemic effects and displayed very high safety indices in nude mice. MT19c is a weak VDR antagonist that disrupts VDR-coactivator interactions and did not exhibit classical calcitriol-VDR interactions [18]. At low doses of MT19c, ovarian xenograft tumors or syngeneic rats showed partial to complete response and extended the survival rate significantly compared to control animals. This study outlines a new approach to design safe and efficacious class of vitamin-d anticancer agents that are devoid of hypercalcemia as well as classical vitamin-D type toxicities. Moreover, data provided herein verified that current emphasis on targeting *de novo* fatty acid synthesis enzyme machinery to treat ovarian cancer is a viable approach to treat human ovarian cancer, and based on this study MT19c has been identified as a promising candidate for clinical evaluation in human ovarian cancer patients.

## Results

### Efficacy studies of MT19c in animal EOC models

**Figure 1:**
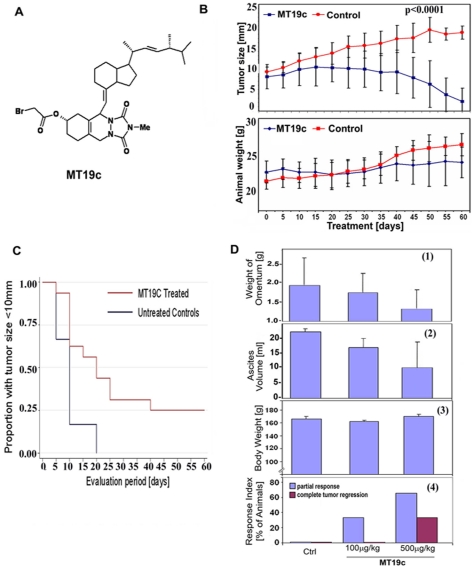
Chemotherapeutic properties of MT19c *in vivo*. (**A**) chemical structure of MT19c. (**B**) **Anti-cancer activity of MT19c in an EOC model in mice.** Nude mice (20 treated and 10 controls) bearing SKOV-3 derived tumor xenografts were dosed (IP) with either vehicle control or MT19c (5 mg/kg bwt) on alternate days for 60 days. Tumor size was calculated (upper panel) using a caliper every 5 days and weight recorded (lower panel). (**C**) **Kaplan-Meier survival analysis.** Kaplan-Meier survival analysis for MT19c and vehicle-treated mice was performed using STATA 9 (StataCorp, College Station, TX) and SAS 9.1 software (SAS Institute, Cary, NC). (**D**) **Efficacy of MT19c in a syngeneic EOC model in rats.** Fisher 344 rats (3 animals/treatment group) were injected IP with rat EOC cells NuTu-19. After 3 weeks, either MT19c (100 or 500 µg/kg bwt) or vehicle were injected IP daily for 12 days. Tumor tissues were harvested and omental weight (D-1), ascitic volume (D-2) and body weight (D-3) recorded. Mean omental weight and volume were compared by Student's T-test with unequal variances. The lower panel depicts the response index (D-4).

The anti-tumor efficacy of MT19c (Fig. 1A) was studied using human EOC cell derived xenografts in nude (NU/NU) mice as well as rat ovarian cancer based syngeneic rat model in Fisher-344 rats. For the first study SKOV-3 cells suspended in matrigel were inoculated subcutaneously in one flank of each animal. Animals were assigned to a treatment (n = 20) or a control group (n = 10). Vehicle or MT19c (5 mg/kg bwt) was administered IP every other day for 60 days to mice bearing SKOV-3 derived tumors. Animals were weighed (Figure 1B, lower panel) and tumor size measured (Figure 1B, upper panel) every 5 days. Normal weight gain for both vehicle and drug treated mice was observed during treatment. Tumor size increased in the control animals with an average 2-fold increase of tumor diameter during the trial period. In the treatment group, tumor size decreased significantly during the last 15 days of treatment with 5 of 8 animals showing complete response (Fig. 1B, upper panel). Animal survival rates were significantly different between treatment and control groups (p = 0.0001, Fig. 1C) based on Kaplan-Meier analysis. During the evaluation period, vehicle treated mice reached the end point (10 mm tumor diameter) within 20 days of treatment whereas a portion of MT19c treated animals survived until study end.

As an independent approach to determine the anti-cancer activity of MT19c *in vivo* we employed a well defined syngeneic rat EOC model [19] Fisher 344 rats were divided in two treatment groups of 3 animals each and one control group. NuTu-19 rat EOC cells were injected IP. After 3 weeks, either 0.1 or 0.5 mg MT19c/kg bwt or vehicle were injected IP daily. An average of 22 ml of ascitic fluid was collected in control animals at study endpoint (Fig. 1D, ascites volume). Multiple small tumors (0.1–1.5 cm in diameter) were observed on the omentum, bowel, diaphragm, peritoneal wall, and surface of all other abdominal organs. MT19c treatment dose-dependently reduced average ascites formation at 0.5 mg/kg bwt and suppressed tumor nodule formation. Similarly, MT19c treatment dose-dependently reduced the average omental weight (Fig. 1D). In this study we used a very low dose of MT19c as compared to the acute toxicity study (400 mg/kg bwt) or the above mice xenograft model and long-term toxicity study (5 mg/kg bwt; 10× greater). Remarkably, at the dose of 0.5 mg MT19c/kg bwt all 3 animals treated showed tumor regression (Fig. 1D, response index). One animal showed a complete response. Animal weights in both control and treatment groups increased during this experiment (Fig. 1D).

### MT19c effects on serum calcium levels and toxicity studies in animal models

**Figure 2:**
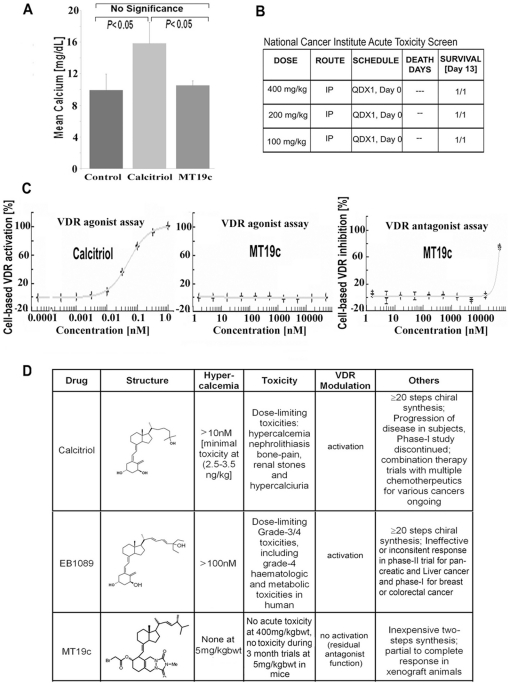
Characteristics of MT19c as a vitamin-D3 derivative. (**A**) **Serum calcium levels in mice after MT19c treatment.** 8 mice each were treated with MT19c(5 mg/kg bwt) or calcitriol (10 ug/kg bwt) or vehicle (EtOH) for 35 days, blood collected and serum calcium analyzed at day 35. Change in mean serum calcium was significant (P<0.05) compared between groups by Student's T-test with unequal variances (**B**) **Acute toxicity study of MT19c**. MT19c or vehicle was administered to nude mice and animals were monitored for any observable toxicity. (**C**) **MT19c in a VDR-agonist or antagonist screening.** VDR-over-expressing VDR-UAS-bla HEK 293T cells were treated for 5 h with calcitriol/vitamin-D3 (0.1 pM-1 nM; left panel) or MT19c (1 nM-1 µM; middle panel) and VDR-activation was analyzed. To analyze antagonistic effects the assay was carried out (SelectScreen® Cell-based Nuclear Receptor Profiling Services: http://www.invitrogen.com) after cell stimulation with calcitriol/vitamin-D3 (120 pM) and treatment with MT19c (1 nM-1 µM; right panel;) for 5 h. (**D**) **Summary of salient features of MT19c and related compounds**. Comparison of MT19c with to calcitriol and other clinically relevant vitamin-d derivatives.

Currently known calcitriol/vitamin-D3 analogs cause hypercalcemia. We therefore investigated if MT19c can cause hypercalcemia in animals despite altered A-ring conformation. In animals, MT19c did not cause hypercalcemia during 35days of animal trial. Calcitriol showed significantly higher serum calcium levels (15.5 mg/dL) at the end of treatment than MT19c treated mice (∼10 mg/dL) which was closer to calcium levels in control group (p<0.05) (Fig. 2A).

An acute toxicity study to determine safety of MT19c was performed by the National Cancer Institute (NCI) in athymic nude mice. MT19c was administered intraperitoneally (IP) at doses 400, 200, 100 mg/kg bwt (one animal/dose) and animals were monitored for a period of 13 days (Fig. 2B) to record any observable toxicity (see Supporting Information S1). MT19c did not show any toxicity at any of three doses tested. Acute toxicity measures the concentration that will adversely affect the animal's health, and lethality is the most common endpoint.

### MT19c does not affect VDR transcription in cells

MT19c showed a weak antagonistic effect in a fluorescence polarization assay using the VDR ligand binding domain and a fluorescent labeled coactivator peptide [20]. To determine transcriptional regulation of VDR in cells upon MT19c treatment we employed a cell-based functional-VDR-reporter assay (GeneBLAzer® Technology, www.invitrogen.com) using transformed HEK293 cells (see Supporting Information S1). These HEK293T cells express a fusion protein of VDR-LBD–GAL4 DNA-binding domain, which is activated by calcitriol and induces transcription of a beta-lactamase reporter gene. The transcriptional activation of VDR in the presence of MT19c was determined after 5 hr pre-treatment with the control calcitriol (0.1 pM–1 nM) (Fig. 2C; left panel) or MT19c (1 nM–1 µM) (Fig. 2C; middle panel). Calcitriol caused VDR-activation at 10 pM (IC_50_∼30 pM). MT19c showed no agonistic activity at the concentrations tested. To analyze antagonistic effects, cells stimulated by calcitriol (120 pM) were treated with MT19c (1 nM–50 µM) (Fig. 2C; right panel) for 5 h. MT19c inhibited calcitriol-induced VDR-activation only at relatively high concentrations (IC_50_∼30 µM). Thus MT19c emerged as an extremely weak VDR antagonist not reaching biological significance. MT19c is approximately 1000 times less potent VDR antagonist than TEI-9647 or ZK159222 [21].

### Molecular Docking Simulation (MDS) of VDR and MT19c interaction

**Figure 3:**
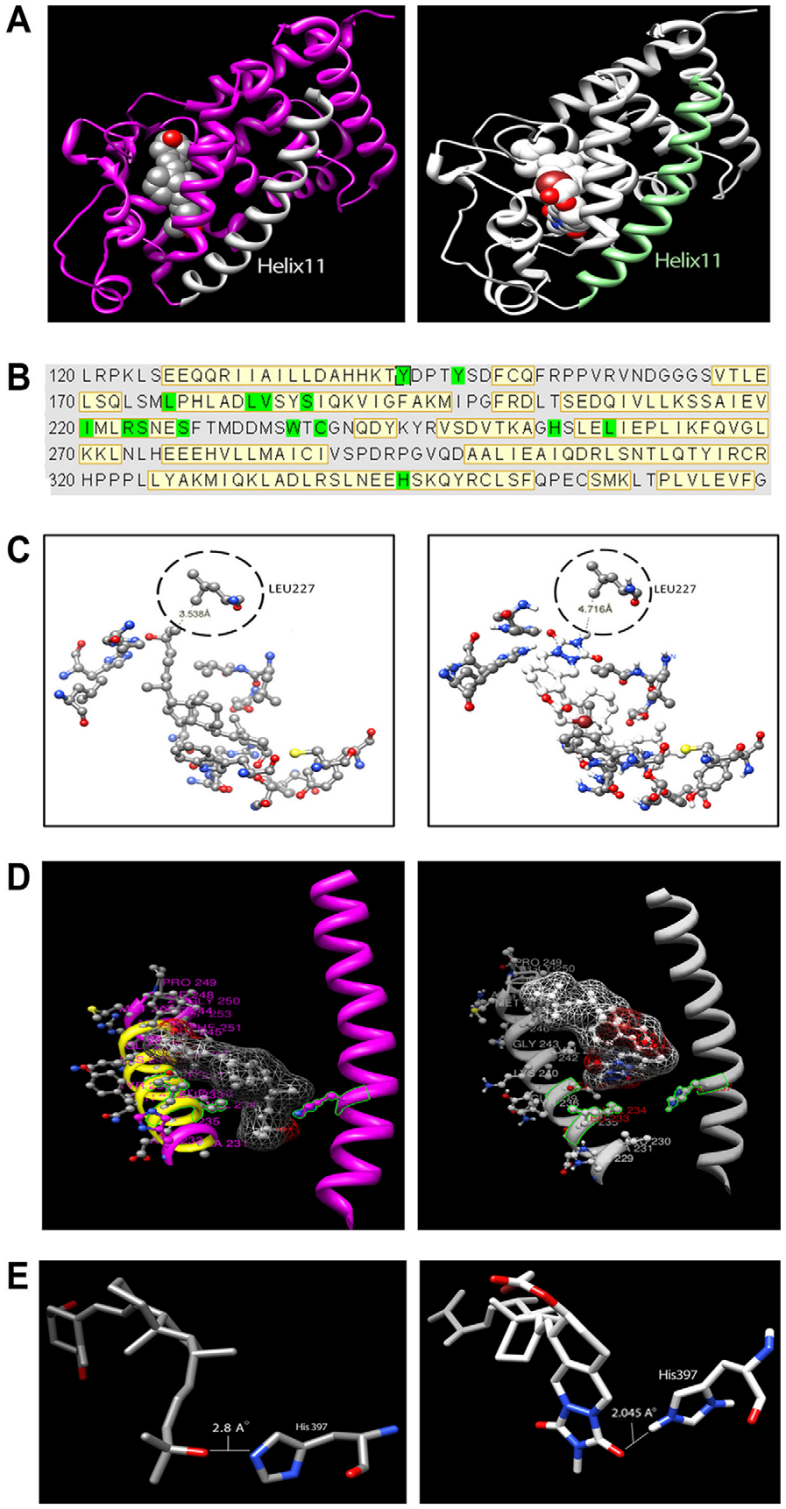
Molecular Docking Simulation of VDR and MT19c. (**A**) **3D structures of VDR/calcitriol and VDR/MT19c complexes.** *Left panel:* VDR/calcitriol complex (Calcitriol in center, helix 11 = white color). *Right panel:* VDR/MT19c complex (MT19c in center, 11 = light green). MDS was carried out using the AutoDock 4.0 program with the structure of MT19 and of calcitriol-liganded VDR provided by the Protein Data Bank. Images of structures were generated using UCSF Chimera. (**B**) **Sequence of VDR ligand binding site**. Yellow color code represents helices in the structure. Green color code represents amino acids with direct interaction to the ligand (calcitriol). (**C**) **Interaction comparison.** *Left panel:* Interaction between Leu227 and calcitriol. *Right Panel:* Interaction between Leu227 and MT19c. (**D**) **Comparison of helix 12 and helix 11 interactions with ligands.** *Left panel:* Interaction between helix 11 (purple), helix 12 (yellow), and calcitriol. *Right Panel:* Interaction between helix 11 (white), helix 12 (yellow) and MT19c. In both panels interaction between His397 on helix 11 and ligand is depicted. (**E**) **Distances between His 397 and ligands.** *Left panel:* Interaction between His397 and calcitriol. *Right Panel:* Interaction between His397 and MT19c.

Most of the currently known calcitriol/vitamin-D3 analogs are VDR agonists. For molecular insight into a possible interaction between MT19c with the VDR we employed a MDS based on the structure of MT19c and the VDR liganded to calcitriol (PDB ID: 1DB1) [22] utilizing the AutoDock 4.0 program [23]. Possible protein-ligand complexes were selected based on their binding free-energy and the conformation with the lowest docked energy was chosen as a possible candidate for MT19c/VDR interaction. Fig. 3A depicts the MDS for VDR and calcitriol (left panel) or MT19c (right panel). In the binding pocket of VDR, the residues that are in contact with calcitriol were used as a reference (Fig. 3B) and for MT19c the same residues were chosen to characterize interactions. We observed that MT19c acquired an inverted accommodation in VDR ligand binding domain compared to calcitriol. MT19c is the first vitamin-D class of molecule to demonstrate this unique VDR-LBD interaction. Up-side accommodation of MT19c in VDR-LBD was highly consequential that disabled the classical calcitriol-VDR like interactions which define the genomic functions of vitamin-D. Further, the key space between helices 11 and 12 in the VDR ligand-binding domain where calcitriol or the antagonist ZK168281 side chain (C-25) bind (Fig. 3D, left panel) is occupied by the A-ring of MT19c (Fig. 3D, right panel). Interestingly, MT19c revealed a closer hydrogen binding between its carbonyl of the A-ring with VDR residue H397 (2.05 Å) (Fig. 3E, right panel) as compared to the 25-hydroxy group of calcitriol (2.8 Å) (Fig. 3E, left panel). Multiple effects described above including the enlarged altered A-ring structure of MT19c that disrupted the natural conformation of helices 11,12 and 13 upon inverted entry in the VDR-LBD, biologically irrelevant interactions with 15 key amino acid residues of VDR-LBD, and distortion in helices 11, 12 and 13 that enables VDR-coactivator interactions may explain why MT19c is non-hypercalcemic in animals [21].

### MT19c inhibits EGFR signaling both in vitro and in vivo but does not affect PPAR-gamma expression

**Figure 4:**
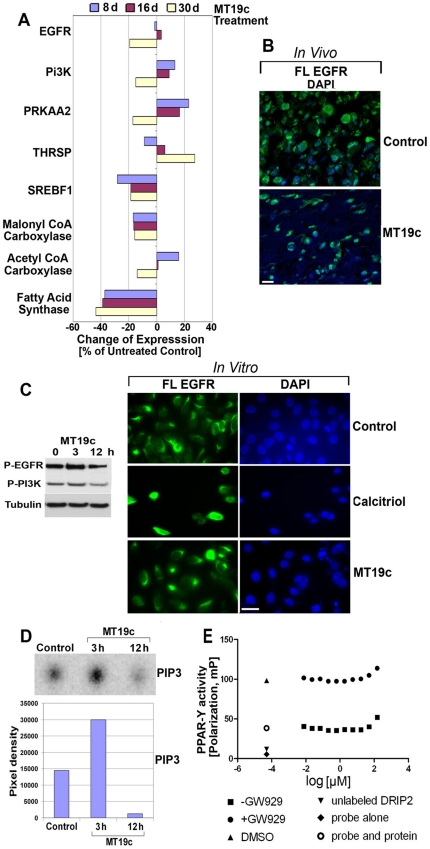
Cellular and biochemical effects in MT19c treated EOC cells *in vitro*. (**A**) **Genomic analysis of naïve or MT19c treated xenograft tumors.** SKOV-3 xenografts were treated with vehicle or MT19c (5 mg/kg bwt) and tumor tissue were harvested on day-8, 16 and 30. The mRNA from tumors was analyzed by Affymetrix microarray chips in triplicate and expression of genes was clustered by GSEA analysis. The difference in expression of the genes from MT19c treated tumors was expressed as % of the control (±). (**B**) **Expression of nuclear EGFR (nEGFR) in SKOV-3 xenograft tissues treated with vehicle or MT19c.** SKOV-3 xenografts were treated with MT19c or vehicle. Paraffin embedded tissues were processed and stained with Alexafluor-conjugated phosphor-EGFR antibody and analyzed by confocal microscopy as described in Material and Method section. EGFR is shown in the green along with nuclear stain DAPI. Magnification: 40×2 (**C**) **Western blot analysis of phospho-EGFR and phospho-PI-3K and expression of nuclear EGFR (nEGFR) in SKOV-3 cells.** (Left panel): SKOV-3 cells were treated with 250 nM MT19c. PAGE and Western blot analysis of cell lysates was carried out. Activated phospho-EGFR and phospho-PI-3K was visualized by immunoblotting using primary antibodies recognizing cleaved fragments. As an internal standard for equal loading (50 µg total cell protein/lane) blots were probed with an (-tubulin antibody. (right panel): expression of nuclear EGFR (nEGFR) in vehicle (upper panel), calcitriol (2 uM, middle panel) and MT19c (250 nM, lower panel) treated SKOV-3 cells by immunofluorescence microscopy. EGFR is shown in green and right columns shows DNA (blue). Magnification: 40. (**D**) **MT19c suppressed PI-3k kinase activity in SKOV-3 cells**. SKOV-3 cells were treated with MT19c (0, 250 nM) for indicated time intervals. Cell lysates were immunoprecipitated with an antibody specific for phospho-tyrosine and PI3K activity was determined with in vitro lipid kinase assay. PIP-3 (phosphoinositide 3-phosphate), the phosphorylated end-product is shown. The bar graph shows the densitometric scanning results of a representative experiment. (**E**): **Identification of interaction between MT19c and PPARγ using fluorescence polarization.** Disruption of binding between PPARγ-LBD and fluorescent DRIP2 peptide by MT19c was investigated in the ▪ presence and • absence of agonist GW929. Controls were vehicle DMSO (▴ with agonist, ○ without agonist), ▾ unlabeled DRIP2 peptide (positive control) and ♦ fluorescent DRIP2 peptide by itself (positive control).

To identify the molecular targets of MT19c in ovarian cancer cells, a Gene Set Enrichment Analysis (GSEA) analysis of the genome wide mRNA of vehicle treated (control) and MT19c treated tumors on day-8, day-16 and day-30 was conducted in triplicates using Affymetrix Human Gene 1.0 ST Array (Affymetrix, Santa Clara, CA). The expression data has been deposited at address www.ncbi.nlm.nih.gov (acc = GSE23616). MT19C treated xenograft tumors consistently showed lower expression levels for genes involved in energy metabolism (p<0.00000000055). Based on expression levels with higher statistical significance (p = 0.00000005; cutoff point), we clustered the key metabolic genes that were affected by an Ingenuity Pathway Analysis (IPA). The differential expression of EGFR, PI3K, PRKAA2, THRSP, SREBF1, Malonyl Co-A carboxylase, Acetyl CoA carboxylase and Fatty acid synthase in control and treatment group on the day-8, day-16 and day-30 is shown in Fig. 4A.

Cancer cells organize the energy requirement by reorienting lipogenic/lipolytic metabolic balance to provide for and sustain the cancer growth by redefining functions of EGFR, PPAR-gamma, and fatty acid synthesis and glycolysis machinery [24]. Targeting EGFR and other components of metabolism has been, therefore, postulated to improve ovarian cancer therapy outcome [6], [25]. To understand the effect of MT19c on EGFR and its downstream signaling cascade in ovarian cancer, we investigated the effect of MT19c on EGFR and PI-3kinase activity in SKOV-3 cells by western blot analysis, microscopy and an *in vitro* PI-3kinase activity assay.

**Figure 5:**
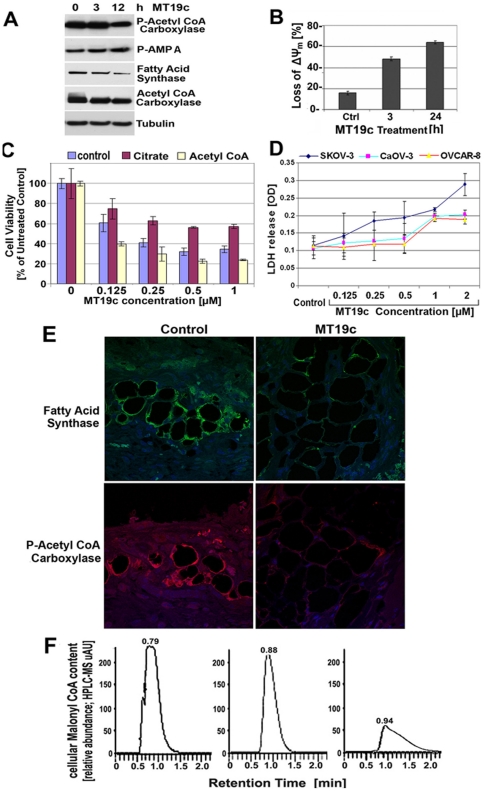
MT19c disrupts mitochondrial functions and fatty acid synthesis machinery in ovarian cancer cells or xenograft tissues. (**A-C**) **Western blot analysis of lipogenetic proteins in SKOV-3 cells.** SKOV-3 cells were treated with 250 nM MT19c or vehicle for 24 h. Analysis of the expression of proteins by western blotting of lysates with primary antibodies against fatty acid synthase (FASN), Acetyl co-A carboxylase (ACC), phosphorylated ACC and AMPA was carried out (Material and Methods). Representative experiments are shown. As an internal standard for equal loading (50 µg total cell protein/lane) blots were probed with an anti-(-tubulin antibody. (**B**) **Mitochondrial transmembrane depolarization-potential (Δ**Ψ**m) analysis after MT19c treatment**. SKOV-3 cells were treated for 3 or 24 h with 1 µM MT19c fixed and stained with DiOC_18_(3) and FACS analysis carried out. The bar diagram depicts the number of non-fluorescing cells (%) with ΔΨm loss. A representative experiment is shown. (**C**) **Effect of fatty acid synthase substrates on cytotoxicity of MT19c.** SKOV-3 cells, preincubated with citrate (1 mM), acetyl co-A (200 µM) for 1 hr and varying concentration of MT19c (0, 1 µM) were added and cells were incubated for 24 hrs and cell viability was determined by MTS assay. (**D**) **LDH release in SKOV-3 cells.** SKOV-3 cells were treated with varying concentration of MT19c (0, 1 µM) were added and cells were incubated for 24 hrs and LDH release estimated using cytotox kit (Promega). (**E**) **expression of fatty acid synthesis proteins in naïve and MT19c treated xenograft tumors.** Expression of Fatty acid synthase (FASN) and phospho-acetyl CoA carboxylase (ACC) in vehicle (left panel) and MT19c (5 mg/kg bwt, right panel) treated SKOV-3 xenografts was determined by a confocal immunofluorescence microscopy. FASN is shown in green and phospho-ACC is shown in red. DNA is shown in blue. Magnification: 40×2. (**F**) HPLC-MS quantification of malonyl-co-A content in MT19c treated SKOV-3 cells. SKOV-3 cells were treated with vehicle or MT19c (500 nM) in serum free DMEM media. Acid soluble extracts were analyzed by HPLC-MS. The area integrals and retention time of the malonyl CoA in control and treatment group was compared to the reference standard (Sigma-Aldrich). (left panel): retention time of reference standard (Sigma Alrich, USA); (middle panel): retention time of malonyl CoA in the vehicle treated SKOV-3 cells; (right panel): retention time of malonyl CoA in the MT19c (500 nM) treated SKOV-3 cells.

Gene expression (GSEA) analysis showed that MT19c suppressed EGFR expression in the tumors treated on the day-8. Moreover, tumors treated up to day-30 showed significant downregulation of EGFR while minor activation of EGFR in tumors treated up to day-16 indicating counteraction effort against the drug action was observed (Fig. 5A). Next, we validated the microarray data on EGFR by immunohistochemical analysis of treated SKOV-3 xenografts treated with vehicle or MT19c (5 mg/kg bwt). The slides of snap frozen harvested tumors from the vehicle or drug treated animals in the Fig. 1B were immuno-stained with an FITC-EGFR antibody (R&D systems, MN, USA) and counter-stained with DAPI dissolved in Vecta-shield mounting media (Vector labs, CA). A confocal microscopy of the phospho-EGFR stained control tumors showed strong cytosolic staining and densely packed nuclei, while MT19c treated tumors demonstrated significantly reduced quantitative staining or expression compared to control (Fig. 4B).

To study the functional effect of MT19c on EGF receptor expression in ovarian cancer cells we examined EGFR localization in SKOV-3 cells *in vitro* upon treatment with MT19c. SKOV-3 cells were treated with MT19c (100 nM) or calcitriol (2 µM) for 12 hr in serum free conditions. The cells were processed as described in Material and Method section. A microscopic examination of the vehicle treated cells showed clear and intact cellular morphology with transmembrane EGFR staining in company with few nuclei densely stained (Fig. 4C, upper left panel). A DAPI staining also showed intact structural integrity of DNA and chromatin (Fig. 4C, upper right panel). Contrary to vehicle, calcitriol treated cells showed highly intense and specific nuclear staining due to nuclear translocalization and demonstrated lack of transmembrane staining (Fig. 4C, middle panels). On the other hand, contrary to calcitriol, MT19c treatment did not promote EGFR nuclear translocalization and a strong transmembrane EGFR staining among cell population similar to vehicle treated cells were observed (Fig. 4C, lower panels). Few nuclei with dense nuclear stain in MT19c treated cell population, in fact, were reminiscent of apoptosis than nuclear translocation. It was noted that xenograft tumors showed stronger cytosolic staining than transmembrane staining observed for cultured SKOV-3 ovarian cancer cells, possibly indicating the differential tumor micro-environment of the both type of cells (Fig. 4B and 4C). A western blot analysis of the drug treated SKOV-3 cells showed that MT19c suppressed EGFR activation within 12 hrs of treatment (Fig. 4C, left panel).

Since EGFR directly regulates many critical functions of PI-3kinase [5], to analyze PI-3kinase activity in SKOV-3 cells upon application of MT19c (250 nM) we performed a PI-3kinase activity [26]. The PI3-kinase (PI3K) pathway regulates many cellular processes such as cell metabolism, cell survival, and apoptosis in cancer and phosphotidylionositol-3,4,5-triphosphate (PIP-3) is the key mediator of PI-3kinse signal transduction [27]. PIP-3 is synthesized from phosphotidylionositol-4,5-diphosphate (PIP-2) [28]. Based on our immunoprecipitation assay, we observed that MT19c treatment (250 nM) downregulated PIP-3 production significantly within 12 hrs compared to control (Fig. 4D). The pixel density measurement of loading spot representing PIP-3 amount of the control lysate eluted via thin layer chromatography (TLC) was 14480 units whereas the MT19c treated sample showed 9 fold less quantitative synthesis of PIP-3 (pixel density 1304) (Fig. 4D, lower panel). Surprisingly, PIP-3 production was strongly upregulated 2-fold within first 3 hours of treatment indicating pro-survival pressure upon the application of the drug. To further understand the effect of MT19c on the expression of PI-3kinase, a Western blot was carried out. MT19c (250 nM) treatment showed downregulation of PI-3kinase phosphorylation in SKOV-3 cells within 12 hours, (Fig. 4C, left panel).

### MT19c does not affect PPAR-gamma component of lipid metabolism in ovarian cancer cells

EGFR overexpression activates PPAR-gamma function in cancer cells to protect them from palmitate toxicity [24]. PPAR-gamma is a potential target for the prevention and treatment of cancer [29]. We examined the interaction of MT19c with nuclear receptor PPAR-gamma by conducting a fluorescence polarization assay. In the absence of PPAR-gamma agonist (GW949) we did not observed an interaction between PPARγ and coactivator protein DRIP2. Similarly, in the presence of agonist GW949 no disruption of the interaction between VDR and DRIP2 was observed.

### MT19c suppressed Fatty acid synthase expression in xenograft tumors

EGFR/PI-3Kinase enhances lipogenesis in cancer cells by activating lipogenic fatty acid synthase (FASN) machinery in conjunction with PPAR γ [5]. Overexpression of FASN in human epithelial ovarian cancer (EOC) has been shown [7]–[10]. An oligonucleotide microarrays screen identified fatty acid synthase (FASN) as potential molecular target in EOC [17]. Genome-wide mRNA analysis of the naïve and MT19c treated xenograft tumors clearly identified MT19c action on FASN expression in MT19c treated ovarian cancer xenograft (p = 0.00000000055) (Fig. 4A) [30].

We observed via immunoblotting that MT19c treatment suppressed expression of FASN and acetyl Co-A carboxylase (ACC) in SKOV-3 cells (Fig. 5A) time dependently. We examined whether MT19c regulates FASN and ACC in treated xenograft tumors. Harvested xenografts of the naïve and MT19c treatment groups were immuno-stained with FASN antibody and analyzed by fluorescence microscopy to distinguish the FASN staining in control group versus treatment group. While vehicle treated tumors showed intense and homogenous staining around lipid droplets embedded in the tissues (Fig. 5D, left upper panel), MT19c treated tumors showed significant lack of staining or partial/residual staining (Fig. 5E, right upper panel). We observed the presence of 2 fatty fields in the whole tissue section that were densely stained in the control. Similarly only two fatty fields were detected in the drug treated xenograft section and the FASN staining around them was significantly lower than vehicle treated control tissues. We carried out fluorescence intensity measurements of whole tissue sections of both control and drug treated tissue and calculated the mean and the Integrated Optical Density (IOD). The fluorescence intensity of the drug treated tissue showed a mean value of 11697 units and 4.14 units of IOD compared to 22805 units of mean and 17.15 units of IOD observed for vehicle treated xenograft tissue indicating significant reduction in staining in MT19c treated xenograft tumors.

Acetyl CoA-carboxylase (ACC) is the rate-limiting enzyme for the long chain fatty acid synthesis and is also a molecular target of much current research interest for the treatment of obesity and cancer [4], [5]. Harvested xenografts of the naïve and MT19c treatment groups were immunostained with phospho-ACC antibody and analyzed by fluorescence microscopy to distinguish the ACC staining in control group versus treatment group. While vehicle treated tumors showed intense staining around lipid droplets embedded in the tissues (Fig. 5E, left lower panel), MT19c treated tumors showed lack of staining or partial/residual staining (Fig. 5D, lower right panel). We noted the presence of four fatty fields in the whole tissue section that were densely stained in the vehicle treated tissue (only one of the fields is shown). However only two fatty fields were detected in the drug treated xenograft section and the p-ACC staining around them was significantly lower than vehicle treated control tissues. The intensity of the drug-treated tissues showed a mean value of 13121 units and 5.54 units of IOD compared to 23438 units of mean and 39.58 units of IOD observed for vehicle treated xenograft tissue indicating significant reduction in staining in MT19c treated xenograft tumors.

### MT19c reduced mitochondrial depolarization potential and citrate homeostasis and causes LDH release in ovarian cancer cells

FASN overexpression protects cells from apoptosis via stabilization of mitochondrial membrane potential [31]. As shown in Fig. 5B MT19c (1 µM) treatment reduced the transmembrane mitochondrial potential of ∼30% of SKOV-3 cell population within 3 hrs of drug treatment and within 24 hrs more than 60% of the cell population had lost membrane depolarization potential. Therefore MT19c treatment significantly disabled mitochondrial health of SKOV-3 cells leading to early cell death observed.

Since enzymes of citric acid cycle are located in the mitochondrial matrix, we examined if citrate catabolism is impaired due to disrupted mitochondria upon treatment with MT19c in SKOV-3 cells. SKOV-3 cells were pretreated with citrate (500 µM) for 1 hr and MT19c (0–1 µM) or vehicle was applied for 24 hrs. While MT19c alone reduced the cell viability of SKOV-3 cells strongly, citrate pretreatment rescued the cells from MT19c induced cytotoxicity significantly even at a highly toxic concentration of MT19c (1 µM) (Fig. 5C). Therefore, MT19c disabled mitochondrial functions and blocked citrate catabolism in the cells depriving the cells of the key building block (i.e. acetyl co-A) for *de novo* fatty acid synthesis.

To determine if MT19c targeted the lactate synthesis machinery of the glycolysis, we quantified the release of lactate dehydrogenase (LDH) in three different EOC cell lines. LDH is a stable cytoplasmic enzyme and cytotoxic agents induce a release of LDH, the rate determining enzyme in the lactate synthesis. We determined the release of LDH by a Cytotox 96® kit (Promega, cat no-G1782). Our experiment revealed that MT19c at concentrations of lower than 1 µM caused significant LDH release within 24 h in SKOV-3, CaOV-3 and OVCAR-8 EOC cells (Fig. 5D). We infer based on LDH release that MT19c did not permit the alternative metabolic pathway that feeds on lactate synthesis, in addition to disrupting mitochondrial function and blocking the fatty acid synthesis machinery.

### MT19c suppressed malonyl CoA synthesis in SKOV-3 cells

Malonyl CoA is the main component that fatty acid synthase converts into palmitates. We investigated if MT19c could restrict the levels of malonyl CoA in ovarian cancer cells upon treatment. Direct measurement of malonyl CoA in SKOV-3 cells by reversed- phase HPLC of acid soluble extracts from vehicle or drug treated cells confirmed that MT19c caused marked decrease in malonyl CoA levels similar to the functions of TOFA a fatty acid synthase inhibitor. Fig. 5F is a representative chromatograph represents reference standard of malonyl CoA. Malonyl CoA is the first of these to elute within 1 minutes. The estimation of the area under curve of control (middle panel) and MT19c treated sample (right panel) shows that MT19c treatment led to a marked decrease (∼60%) in malonyl CoA content in the drug treated SKOV-3 cells compared to the untreated cells within 3 hours of the treatment. The chemical identity of the malonyl-CoA was independently confirmed by comparing the retention time (left panel) and mass spectrum of malonyl-CoA (reference standard from Sigma-Aldrich, USA) using a UV detector at 254 nM.

## Discussion

Calcitriol and analogs displayed anti-tumor effects against various cancer types [32]. However, in clinical trials calcitriol and analogs displayed the lack of efficacy and caused hypercalcemia, hyperphosphatemia, and secondary effects such as vascular calcification, nephrocalcinosis or adynamic bone disease [32 and references cited therein]. These adverse outcomes have prompted the development of less hypercalcemic and more efficacious vitamin-D analogs. We developed a new approach to design vitamin-D based molecules that are amenable to small molecule library synthesis and subsequent high-throughput screening. MT19c was designed by a targeted chemical modification of A-ring of vitamin-D2 in just two efficient steps, in contrast to the elaborated synthetic efforts needed for calcitriol or EB1089. We incorporated a nitrogen- and oxygen-rich heterocyclic triazolinedione ring to balance the structure of the molecule in terms of Lipinski's rule [33]. In a preliminary study, we showed anti-cancer actions of MT19c in cultured ovarian cancer cell models [20].

The present study shows that the novel compound MT19c is not only the first true non-hypercalcemic vitamin-D derivative but also reveals promising activities as an anti-tumor agent in various EOC animal models. *In vivo*, MT19c treatment at 5 mg/kg bwt did not elevate serum-calcium levels and showed no acute toxicity even at 400 mg/kg bwt. In human EOC derived xenografts the majority of treated mice displayed complete response and extended tumor free survival significantly (p<0.0001). In a syngeneic rat model all animals treated with MT19c even at a low concentration (100–500 µg/kg bwt) showed tumor regression and absence of ascites formation, and cleared lesions in peritoneum and omentum.

MT19c did not cause hypercalcemia in animals even at 500× dose of calcitriol. A VDR trans-activation and a VDR receptor binding assay revealed that in contrast to calcitriol, MT19c showed a biologically inconsequential weak VDR antagonism. Lack of MT19c-VDR agonistic interaction was further supported by the in silico molecular docking simulations performed for MT19c with VDR crystal structure. Due to lower energy upside-down docking of the bulkier and rigid MT19c in VDR-LBD, 15 amino acid interactions with MT19c are altered in comparison with the structure of classical calcitriol-VDR interactions. Furthermore, the docked structure of MT19c-VDR-LBD has a altered position of helix 12 and 13, which is essential for the interaction with coactivators. This behavior has been observed for other VDR antagonists [21].

To identify the molecular target and unravel the mechanism of action of MT19c in ovarian cancer models, we previously conducted a genome wide mRNA analysis of the drug treated or naïve tumors at three different treatment points (www.ncbi.nlm.nih.gov (acc = GSE23616). A GSEA analysis to cluster genes identified that lipid metabolism, molecular transport and small molecule biochemistry genes such as ACACA, EGFR, FASN, PRKAA2, PTEN, SCAP, SREBF1IPA involved in lipid synthesis were most significantly downregulated (p = 0.00000000055) (Fig. 4A). Remarkably, these metabolic genes are implicated in understanding the etiology of ovarian cancer and the development of targeted therapies against these growth factors (e.g. EGFR), to improve chemotherapy outcomes in ovarian cancer patients.

Epidermal growth factor receptor (EGFR) overexpression is associated with poor prognosis, resistance to chemotherapy and low survival rate in ovarian cancer [34]. EGFR enhances DNA repair via catalytic subunit of DNA protein kinase (DNA-PKCs) and promotes oncogenic signaling via PI-3K/AKT [35].Targeting EGFR using pharmacological inhibitors such as Iressa has been postulated to improve ovarian cancer therapy outcome and Iressa responding patient population has been identified [36]. In our experiment, fluorescence confocal microscopy showed that MT19c treatment significantly inhibited EGFR activation in ovarian xenograft tumors in animals compared to control (Fig. 4B). Similarly, MT19c treatment did not induce EGFR translocation, or phosphorylate EGFR in SKOV-3 cells (Fig. 4C)

EGFR activation leads to activation of PI-3kinase, via PDK1 and phosphorylation of S473 on the PKB/Akt that inhibits the activity of pro-apoptotic proteins such as, Caspase-9 and GSK-3β [37]–[38] Dysregulation of the PI-3kinase (PI3K) pathway has been implicated in many human diseases and hyperactivation of this pathway promotes tumorigenesis and chemoresistance [38]. Phosphoinositide 3,4,5,-trisphosphate (PIP3) is the main product of PI3K activity. Small molecule antagonists of PIP3–PH domain interactions (e.g. PITenins, PITs) suppressed the PI3K-PDK1-Akt pathway, which triggers metabolic stress leading to apoptosis, and validated the rationale of targeting PIP-3 signaling to inhibit tumorigenesis [37], [38]. MT19c inhibited not only PI-3kinase phosphorylation, it also inhibited the synthesis of PIP-3 the main product of PI-3k activity.

To exert an oncogenic impact, EGFR over-expression directs fatty acid synthesis enzymes (FASN and ACC) to facilitate aerobic glycolysis and ignore oxidative phosphorylation for energy production [6]. The endogenous fatty acid (FA) biogenesis, catalyzed by FASN and its pathway components constitute a major oncogenic stimulus that drives normal epithelial cells progression towards malignancy [39]. Based on overactive FASN, precursor lesions develop an adaptive metabolic response to microenvironments [6]. The oncogenic action of FASN depends on the oncogene EGFR, which induces FASN hyperactivation in a bi-directional manner: i) maintaining incessant de novo fatty acid synthesis via FASN that awards lipogenic and micro-environmental metabolic adaptive benefits to pre-lesion oncogenesis, chemoresistance and metastasis in ovarian cancer [40]–[41]; ii) averting cellular toxicity arising due to end product of FASN action i.e. palmitic acid by conversion of excess fatty acids (FA) to triglycerides in PPAR-gamma dependent manner. MT19c treatment downregulated FASN and ACC activation both in *in-vitro* and *in-vivo* models of ovarian cancer. Abrogation of de novo lipogenesis by MT19c occured PPAR-gamma independently.

The inhibition of fatty acid synthesis and the activity of FASN and ACC are directly linked to the repudiation of citrate homeostasis in mitochondria [5], [6]. In tumor cells, mitochondria accumulate lipophilic cations that increases their membrane potential. The reduction of this increased membrane potential either by chemotherapeutics or by stress stimuli causes cell cycle arrest and apoptosis [42]. MT19c disrupted the mitochondrial transmembrane depolarization potential within 3 hours of the treatment indicating that citrate homeostasis was disrupted either prior to fatty acid synthesis or concomitantly. Citrate is the mitochondrial substrate that is converted to acetyl CoA catabolically and the mitochondrial disruption by cytotoxic agents (e.g. MT19c ) may reduce the supply of citrate, the key building block for long chain fatty acid synthesis. MT19c inhibition of mitochondrial function and citrate catabolism was directly proven by reduction in cytotoxicity of MT19c upon replenishing the SKOV-3 cells with citrate post MT19c treatment but not with acetyl Co-A, malonyl Co-A or recombinant Acetyl Co-A carboxylase enzyme. MT19c treatment also caused LDH release and blocked lactate synthesis in SKOV-3, CaOV-3 and OVCAR-8 cells. LDH release is a direct measure of a cytotoxic response of chemotherapeutic drugs. Further the direct measurement of malonyl CoA derivatives in SKOV-3 cells by reversed- phase HPLC of acid soluble extracts from vehicle or drug treated cells confirmed that MT19c caused marked decrease in malonyl CoA levels similar to the functions of TOFA a fatty acid synthase inhibitor [43].

In conclusion, we have described a new design approach to non-hypercalcemic vitamin-D derivatives bestowed with potent anticancer efficacy without any toxicity. MT19c is the first non-hypercalcemic vitamin-D derivative that revealed promising anti-cancer efficacies in two independent EOC animal models. MT19c targets the cancer cell metabolism machinery, specifically FASN functions and abrogates *de novo* lipogenesis, the hallmark of cancer cells. This study therefore provides the rationale for developing novel vitamin-D analogs that target *de novo* fatty acid synthesis in ovarian cancer.

## Materials and Methods

### Cell culture and cytotoxicity assay

SKOV-3, OVCAR-3 and OVCAR-8 cells were purchased from American Tissue Culture Collection (ATCC) (www.atcc.org) and maintained as described previously [44]. No de novo cell lines were developed for this study. Quantification of MT19c induced cytotoxicity to a panel of ovarian cancer cell-lines (SKOV-3, OVCAR-3 and OVCAR-80) was determined by a Cytotox 96® cytotoxicity assay kit (Promega, Madison, WI, USA). Experiments were performed in triplicates; data are expressed as the mean of the triplicate determinations (X±SD) of a representative experiment in % of absorbance by samples with untreated cells ( = 100%).

### Hypercalcemia and acute toxicity determination in mice

All animal experiments, with the exception of acute toxicity experiments, were performed in the animal facilities of Rhode Island Hospital (RIH) with strict adherence to the guidelines of the Animal Welfare Committee of RIH and Women & Infants Hospital (WIH) in accordance with the guidelines set by the NIH in the care and use of laboratory animals (Laboratory Animal Protection Approval: A3922-01; CMTT: 0061-07). The Animal welfare committee (RIH and WIH) specifically approved this study.

Thirteen six-week old female nude mice (nu/nu strain code 088/homozygous, 25 g average weight) (Charles River Laboratories, Wilmington, MA, USA) were randomly assigned to a control group (8 animals) or treatment group (8 animals each for MT19c and calcitriol). Vehicle (PBS/2.5% EtOH) or MT19c at 5 mg/kg bwt or calcitriol (10 µM) in 0.2 ml of vehicle was administered intraperitoneally (IP) every other day for 35 days. Animal were monitored daily and weights were measured biweekly. Blood samples were collected on the day 35 by cardiac puncture (endpoint). Calcium concentration analysis was performed by IDEXX Laboratories Inc. (North Grafton, MA).

The acute toxicity study (400, 200, 100 mg/kg bwt MT19c, IP, day 0, one mouse each) was performed by the National Cancer Institute (NCI) Developmental Therapeutics Program (DTP) (www.dtp.nci.nih.gov, athymic nude mice). For details of the method refer Supporting Information S1.

### EOC Xenograft Model in mice

Animal experiments were carried out in the animal facilities of Rhode Island Hospital (RIH), RI, USA with strict adherence to the guidelines of the Animal Welfare Committee of Rhode Island Hospital (RIH) and Women and Infants Hospital of Rhode Island (Laboratory Animal Protection Approval: A3922-01; CMTT: 0061-07). Four to six week-old immunodeficient nude mice (NU/NU; strain code 088/homozygous) (Charles River Laboratories, Wilmington, MA) were maintained at a temperature of 22±1°C and a relative humidity of 55±5%, with a 12 h light/dark cycle. SKOV-3 cells were cultured to 80% confluence, washed in PBS twice, harvested by trypsination, pooled in complete medium, washed in PBS twice, and 2×10^6^ cells/inoculate were suspended in 0.1 ml of matrigel and inoculated subcutaneously in the flank of mice. Mice with developing tumors after two weeks were randomly assigned to experimental groups. MT19c was prepared as a stock solution of 1 mM in 100% EtOH and diluted 1∶40 in PBS for administration. Mice were treated intraperitoneally every other day with either vehicle control (control group; 8 animals) or 300 µl (5 mg/kg bwt) of MT19c (n = 20) for 60 days. Mice were weighed and tumor size calculated using a caliper every 5 days. Survival curves were estimated using Kaplan-Meier method.

### Evaluation of efficacy of MT19c in a syngeneic EOC model in rats

Eight week-old female rats (Fisher 344, average weight 140 g; Charles River Laboratories, Canada) were divided in two treatment groups of 3 animals each (100 µg/kg bwt, n = 3, and 500 µg/kg bwt, n = 3) and one control group (n = 12). Rat EOC NuTu-19 cells (5×10^6^ cells/inoculate) were suspended in PBS and injected IP. Rats were monitored and after 3 weeks treated IP every day with either vehicle (PBS/2.5% EtOH) control or 500 µl (100 or 500 µg/kg bwt) MT19c for 12 days before animals were euthanized, tumor tissues harvested, omental weight and hemorrhagic ascites volume were recorded.

### Structural Modeling and Molecular Docking Simulation (MDS)

MDS was carried out using the AutoDock 4.0 program with the structure of MT19c and calcitriol-liganded VDR (PDB ID: 1DB1) provided by the Protein Data Bank [22]. For MT19c docking simulation the ligand in the binding pocket of VDR was removed. MDS was carried out applying the Lamarckian genetic algorithm [23]. A population size of 150 and 2,500,000 energy evaluations were used for 50 local search runs. The docking area was defined by a box, with grid spacing of 0.375 Å and the dimension of 50×50×50 points along the x, y and z axes. The conformation with the lowest docked energy was chosen as a possible candidate for MT19c. Images of structures were generated using UCSF Chimera.

### Analysis of VDR-modulation by MT19c

Effect of MT19c on VDR was examined using a commercially available VDR transcription assay GeneBLAzer (Invitrogen). Experimental details of this assay are provided in the Supporting Information S1.

### Determination of agonistic and antagonistic properties of MT19c using a PPAR-gamma–coactivator binding assay

The assay has been described in detail previously [45]. Briefly, pET15b-PPARγ-LBD expression plasmid, encoding the PPARγ-LBD (amino acids 173–475) was a generous gift from Gabor J. Tigyi (University of Tennessee, Memphis). PPARγ-LBD was expressed in BL21 (DE3) (Invitrogen), purified by affinity chromatography, and stored at −80°C in buffer (50 mM Tris (pH 8.0), 25 mM KCl, 2 mM DTT, 10% glycerol, 0.01% NP-40). For the assay, MT19c were serially diluted in DMSO and 100 nl of each concentration was transferred into 20 µL protein buffer (20 mM TRIS (pH 7.5), 100 mM NaCl, 0.01% NP-40, 2% DMSO, 10 nM DRIP2 (CNTKNHPMLMNLLKDNPAQD) labeled with Texas-Red maleimide, and 1 µM PPARγ-LBD) in the presence and absence of rosiglitazone (5 µM) in quadruplet using black 384 well plate (Costar, #3658) (see Supporting Information S1). The samples were allowed to equilibrate for two hours. Binding was then measured using fluorescence polarization (excitation 595 nm, emission 615 nm) using a M1000 plate reader (Tecan). The experiments were evaluated using GraphPad Prism 5, and IC50 values were obtained by fitting the data to an equation (Sigmoidal dose-response-variable slope (four parameters). Values are given as the mean values of two independent experiments with a 95% confidence interval.

### Immunohistochemical analysis of tumors

Immunohistochemical staining was performed on paraffin-embedded slides of the vehicle or drug treated tumor specimens (thickness 5 µm). Tissue sections were deparaffinized and rehydrated with serial ethanol dilutions of 100, 95 and 70%. Heat-induced antigen retrieval was then performed using DAKO Antigen Retrieval Solution for 20 min. Tissue sections were blocked with Normal Goat Blocking Serum (Vector Laboratories) for 60 min at room temp before incubating with primary antibodies for phospho-EGFR (R and D systems, MN, USA) (1∶50 dilution), phospho-actetyl CoA carboxylase (1∶200), and fatty acid synthase (1∶200) (Cell Signaling Technologies) in a humidified chamber overnight at 4°C. Secondary antibodies (DyLight 594 goat anti-rabbit IgG, Jackson ImmunoResearch Laboratories, INC. and Alexa Fluor 594 goat anti-mouse IgG at 1∶500, Invitrogen) were applied and incubated for 60 min for 1 hour at room temperature in the dark. Vectashield media with DAPI (Vector Laboratories) was used to mount cover-slips for further analysis. Sixteen bit images were acquired with a Nikon E800 microscope (Nikon Inc. Mellville NY) using a 40× PlanApo objective. A Spot II digital camera (Diagnostic Instruments, Sterling Heights MI) was used to acquire the images (see Supporting Information S1). The cameras built-in green filter was used to increase image contrast. Camera settings were based on the brightest slide. All subsequent images were acquired with the same settings. Image processing and analysis was performed using iVision (BioVision Technologies, version 10.4.11, Exton, PA.) image analysis software. Positive staining was defined through intensity thresholding and integrated optical density (IOD) was calculated by examining the thresholded area multiplied by the mean. All measurements were performed in pixels. Confocal images were acquired with a Nikon C1si confocal (Nikon Inc. Mellville NY.) using diode lasers 402, 488 and 561. Serial optical sections were performed with EZ-C1 computer software (Nikon Inc. Mellville, NY). Z series sections were collected at 0.3 µm with a 40× PlanApo lens and a scan zoom of 2. The gain settings were based on the brightest slide and kept constant between specimens. Deconvolution and projections were done in Elements (Nikon Inc. Mellville, NY) computer software.

### Mitochondrial transmembrane-depolarization potential assay

SKOV-3 cells (1×10^6^) were seeded in a 100 mm^2^ petri-dish and treated with vehicle (EtOH) or MT19c (1 µM) for 3 or 24 h and the assay carried out as described earlier [44].

### Microarray data

To identify the molecular targets of MT19c in ovarian cancer cells, a Gene Set Enrichment Analysis (GSEA) analysis of the genome wide mRNA of vehicle treated (control) and MT19c treated tumors on day-8, day-16 and day-30 was conducted in triplicates using Affymetrix Human Gene 1.0 ST Array (Affymetrics, Santa Clara, CA). Microarray data is MIAME compliant. The raw expression data has been deposited at address: www.ncbi.nlm.nih.gov (acc = GSE23616)

### Western blot analysis

SKOV-3 cells were seeded into 6-well plates (3×10^5^cells/dish) before treatment with MT19C (1 µM). Preparation of cell lysates, PAGE and immunoblotting with appropriate antibodies purchased from Cell Signaling technology (Beverly, MA, USA) was carried out as described previously [44]

### HPLC quantification of malonyl CoA in SKOV-3 cells

SKOV-3 cells seeded in 100 mm^2^ dishes (3×10^6^cells/dish) were treated with either vehicle or MT19c (250 nM) in serum free conditions. Cellular content of malonyl co-A was analyzed by HPLC following the literature procedure [43] described briefly in the Supporting Information S1.

### Statistical Analyses

Data analysis was performed with STATA 9 (StataCorp, College Station, TX, USA) and SAS 9.1 (SAS Institute, Cary, NC, USA). Two-tailed p-values were presented, with p<0.05 considered statistically significant. Means were compared by Student's T-test with adjustment for unequal variances as appropriate. Tumor growth rates in the MT19c-treated mice and controls were analyzed by repeated measures linear regression. A first-order autoregressive covariance pattern was used to model the within-subject correlation. Treatment group and evaluation days were entered as factors in model along with their interaction term. Tumor growth was also examined by Kaplan-Meier analysis and the log-rank test (see Supporting Information S1). The outcome was growth of 10 mm or more, and follow-up was censored at the end of evaluation (60 days) or at the time of euthanasia. The proportional hazards assumption was checked by graphical inspection.

## Supporting Information

Supporting Information S1 (DOC)

## References

[1] Leitao MM, Hummer A, Dizon DZ, Aghajanian C, Hensley M (2003). Platinum retreatment of platinum-resistant ovarian cancer after nonplatinum therapy.. Gynecol Oncol.

[2] Kuhajda FP (2000). Fatty acid synthase and human cancer: new perspectives on its role in tumor biology.. Nutrition.

[3] Menendez JavierA, Lupu Ruth (2007). Fatty acid synthase and the lipogenic phenotype in cancer pathogenesis.. Nature Reviews Cancer.

[4] Warburg O (1956). On the origin of cancer cells.. Science.

[5] Kuhajda FP (2006). Fatty acid synthase and cancer: new application of an old pathway.. Cancer Res.

[6] Uddin S, Jehan Z, Ahmed M, Alyan A, Al-Dayel F (2011). Over expression of fatty Acid synthase in middle eastern epithelial ovarian carcinoma activates AKT and its inhibition potentiates cisplatin induced apoptosis.. Mol Med 2011;.

[7] Pizer ES, Wood FD, Heine HS, Romantsev FE, Pasternack GR, Kuhajda FP (1996). Inhibition of fatty acid synthesis delays disease progression in a xenograft model of ovarian cancer.. Cancer Res.

[8] Ueda SM, Yap KL, Davidson B, Tian Y, Murthy V (2010). Expression of fatty acid synthase depends on NAC1 and is associated with recurrent ovarian Serous Carcinomas.. J Oncol.

[9] Milgraum LZ, Witters LA, Pasternack GR, Kuhajda FP (1997). Enzymes of the fatty acid synthesis pathway are highly expressed in in-situ breast carcinoma.. Clin Cancer Res.

[10] Pizer ES, Pflug BR, Bova GS, Han WF, Udan MS, Nelson JB (2001). Increased fatty acid synthase as a therapeutic target in androgen-independent prostate cancer progression.. Prostate.

[11] Rashid A, Pizer ES, Moga M, Milgraum LZ, Zahurak M (1997). Elevated expression of fatty acid synthase and fatty acid synthetic activity in colorectal neoplasia.. Am J Pathol.

[12] Cerne D, Zitnik IP, Sok M (2010). Increased fatty acid synthase activity in non-small cell lung cancer tissue is a weaker predictor of shorter patient survival than increased lipoprotein lipase activity.. Arch Med Res.

[13] Sehdev AS, Kurman RJ, Kuhn E, Shih IeM (2010). Serous tubal intraepithelial carcinoma upregulates markers associated with high-grade serous carcinomas including Rsf-1 (HBXAP), cyclin-E and fatty acid synthase.. Mod Pathol.

[14] Uddin S, Siraj AK, Al-Rasheed M, Ahmed M, Bu R (2008). Fatty acid synthase and AKT pathway signaling in a subset of papillary thyroid cancers.. J Clin Endocrinol Metab.

[15] Welsh JB, Zarrinkar PP, Sapinoso LM, Kern SG, Behling CA (2001). Analysis of gene expression profiles in normal and neoplastic ovarian tissue samples identifies candidate molecular markers of epithelial ovarian cancer.. PNAS.

[16] Zhou W, Han WF, Landree LE, Thupari JN, Pinn ML (2007). Fatty acid synthase inhibition activates AMP-activated protein kinase in SKOV3 human ovarian cancer cells.. Cancer Res.

[17] Menendez JA, Vellon L, Mehmi I, Oza BP, Ropero S (2004). Inhibition of fatty acid synthase (FAS) suppresses HER2/neu (erbB-2) oncogene overexpression in cancer cells.. PNAS.

[18] Lempiäinen H, Molnár F, Macias Gonzalez M, Peräkylä M (2005). Antagonist- and inverse agonist-driven interactions of the vitamin D receptor and the constitutive androstane receptor with corepressor protein.. Mole Endocrinology.

[19] Rose GS, Tocco LM, Granger GA, DiSaia PJ, Hamilton TC (1996). Development and characterization of a clinically useful animal model of epithelial ovarian cancer in the Fischer 344 rat.. Am J Obst Gyn.

[20] Brard L, Lange TS, Robison K, Kim KK, Ara T, McCallum MM, Arnold LA, Moore RG, Singh RK (2011). Evaluation of the first Ergocalciferol-derived, non hypercalcemic anti-cancer agent MT19c in ovarian cancer SKOV-3 cell lines.. Gynecol Oncol.

[21] Bury Y, Stienmeyer A, Carlberg C (2000). Structure-activity relationship of carboxylic ester antagonists of the vitamin D3 receptor.. Mol Pharmacol.

[22] Berman HM, Westbrook J, Feng Z, Gilliland G, Bhat TN (2000). The Protein Data Bank.. Nucleic Acids Res.

[23] Morris GM, Goodsell DS, Halliday RS, Huey R, Hart WE (1998). Automated docking using Lamarckian genetic algorithm and an empirical binding free energy function.. J Comput Chem.

[24] Menendez JavierA (2010). Fine-tuning the lipogenic/lipolytic balance to optimize the metabolic requirements of cancer cell growth: Molecular mechanisms and therapeutic perspectives.. Biochimica et Biophysica Acta.

[25] Sewell JM, Macleod KG, Ritchie A, Smyth JF, Langdon SP (2002). Targeting the EGF receptor in ovarian cancer with the tyrosine kinase inhibitor ZD 1839 (‘Iressa’).. Br J of Cancer.

[26] Yano N, Ianus V, Zhao TC, Tseng A, Padbury JF (2007). A novel signaling pathway for α-adrenergic receptor-mediated activation of phosphoinositide 3-kinase in H9c2 cardiomyocytes.. Am J Physiol Heart Circ Physiol.

[27] Franke TF, Kaplan DR, Cantley LC, Toker A (1997). Direct regulation of the Akt proto-oncogene product by phosphatidylinositol-3,4-bisphosphate.. Science.

[28] Vivanco I, Sawyers CL (2002). The phosphatidylinositol 3-Kinase AKT pathway in human cancer.. Nat Rev Cancer.

[29] Merida I, Avila-Flores A (2006). Tumor metabolism: new opportunities for cancer therapy.. Clin Transl Oncol;.

[30] Stuckey A, Fischer A, Miller DH, Hillenmeyer S, Kim KK, Ritz A (2011). Integrated genomics of ovarian xenograft tumor progression and chemotherapy response.. BMC Cancer.

[31] Migita T, Ruiz S, Fornari A, Fiorentino M, Priolo C (2009). Fatty acid synthase: a metabolic enzyme and candidate oncogene in prostate cancer.. J Natl Cancer Inst.

[32] Deeb KK, Trump DL, Johnson CS (2007). Vitamin-D signaling pathways in Cancer: potential for anti-cancer therapeutics.. Nat Rev Cancer.

[33] Lipinski CA, Lombardo F, Dominy BW, Feeney PJ (2001). Experimental and computational approaches to estimate solubility and permeability in drug discovery and development settings.. Adv Drug Del Rev.

[34] Phelps SLB, Schorge JO, Peyton MJ, Shigematsu H, Xiang Li-Lin (2008). Implications of EGFR inhibition in ovarian cancer cell proliferation.. Gyn Onc.

[35] Liccardi G, Hartley JA, Hochhauser D (2011). EGFR nuclear translocation modulates DNA repair following cisplatin and ionizing radiation treatment.. Cancer Res.

[36] Schilder R, Chen X, Armstrong BA, Sill MW, Darcy KM (2005). EGFR mutations in ovarian cancer correlate with response to gefitinib in a phase II trial of relapsed, persistent ovarian or primary peritoneal carcinoma: A gynecologic oncology group study. clinical research 17: therapy and biomarker evaluations of phase II clinical trials.. Proc Amer Assoc Cancer Res.

[37] Cantley LC (2002). The phosphoinositide 3-kinase pathway.. Science.

[38] Miao B, Skidan I, Yang J, Lugovskoy A, Reibarkh M (2010). Small molecule inhibition of phosphatidylinositol- 3,4,5-triphosphate (PIP3) binding to pleckstrin homology domains.. PNAS.

[39] Menendez JA, Decker JP, Lupu R (2005). In support of fatty acid synthase (FAS) as a metabolic oncogene: extracellular acidosis acts in an epigenetic fashion activating FAS gene expression in cancer cells.. J Cell Biochem.

[40] Kumar-Sinha C, Ignatoski KW, Lippman ME, Ethier SP, Chinnaiyan AM (2003). Transcriptome analysis of HER2 reveals a molecular connection to fatty acid synthesis.. Cancer Res.

[41] Grunt TW, Wagner R, Grusch M, Berger W, Singer CF (2009). Interaction between fatty acid synthase- and ErbB-systems in ovarian cancer cells.. Biochem Biophys Res Commun.

[42] Dorward A, Sweet S, Moorehead R, Singh G (1997). Mitochondrial contributions to cancer cell physiology: redox balance, cell cycle, and drug resistance.. J of Bioenerg and biomemb.

[43] Pizer ES, Thupari J, Han WF, Pinn ML, Chrest FJ (2000). Malonyl-Coenzyme-A is a potential mediator of cytotoxicity induced by fatty-acid synthase inhibition in human breast cancer cells and xenografts.. Cancer Res.

[44] Lange TS, McCourt C, Singh RK, Kim KK, Singh AK (2009). Apoptotic and chemotherapeutic properties of iron (III)-salophene in an ovarian cancer animal model.. Drug Des Devel Ther.

[45] Feau C, Arnold LA, Kosinski A, Zhu F, Connelly M (2009). Novel flufenamic acid analogues as inhibitors of androgen receptor mediated transcription.. ACS Chem Biol.

