# Supplementary material for: Efficacy of a Non-Hypercalcemic Vitamin-D2 Derived Anti-Cancer Agent (MT19c) and Inhibition of Fatty Acid Synthesis in an Ovarian Cancer Xenograft Model
Source: PLoS One. 2012 Apr 3;7(4):e34443. doi: 10.1371/journal.pone.0034443 (PMC3317945; doi:10.1371/journal.pone.0034443)
Supplement: Supporting Information S1 — (DOC) [file pone.0034443.s001.doc]

**Supporting Information S1**

# **Efficacy of a non-hypercalcemic vitamin-D2 derived anti-cancer agent (MT19c) and inhibition of fatty acid synthesis in an ovarian cancer xenograft model.**

# Richard G. Moore^1^, Thilo S. Lange^1^, Katina Robinson^1^, Kyu K. Kim^1^, Alper Uzun^2^, Timothy C. Horan^1^, Nada Kawar^1^, Naohiro Yano^3^, Sharon R. Chu^4^, Quanfu Mao^4^, Laurent Brard^5^, Monique E. DePaepe^4^, James F. Padbury^2^, Leggy A Arnold^6^, Tun-Li Shen^7^, Rakesh K. Singh^1^,*

^1^Molecular Therapeutics Laboratory, Program in Women's Oncology, Department of Obstetrics and Gynecology, Alpert Medical School, Brown University, Providence, RI 02905, USA

^2^Center for Computational Molecular Biology, Department of Pediatrics, Women and Infants Hospital, Alpert Medical School, Brown University, Providence, RI 02905, USA

^3^Department of Pediatrics, Women and Infants Hospital, Alpert Medical School, Brown University, Providence, RI 02905, USA

^4^Developmental Pathology, Women and Infants’ Hospital, Program in Women's Oncology and Alpert Medical School, Brown University, Providence, RI 02905, USA

^5^Present address: Gynecology Oncology, Southern Illinois Medical School, Springfield. IL, USA.

^6^Chemistry and Biochemistry, University of Wisconsin-Milwaukee, Milwaukee, WI 53551, USA

^7^Department of chemistry, Brown University, Providence, RI, USA

*Corresponding Author: Rakesh K. Singh MPhil, PhD. Program in Women’s Oncology, Department of Obstetrics and Gynecology, Women and Infants Hospital of RI, 101 Dudley Street, Providence, RI 02905, Tel: 401-274-1122 x 8062, Fax: 401-277-3617, Email: [rsingh@wihri.org](mailto:rsingh@wihri.org).

Short Title: efficacy of MT19c in ovarian cancer models.

**Acute Toxicity Assay**

**A** single mouse is given a single injection (IP, IV, SC, IM or PO) of 400 mg/kg; a second mouse receives a dose of 200 mg/kg and a third mouse receives a single dose of 100 mg/kg. The mice are observed for a period of 2 weeks. They are sacrificed if they lose more than 20% of their body weight or if there are other signs of significant toxicity. If all 3 mice must be sacrificed, the next 3 dose levels (50, 35 and 12.5 mg/kg) are tested in a similar manner. This process is repeated until a tolerated dose is found. This dose is then designated the MTD and is used to calculate the amount of material administered to mice during anti-tumor testing. The mice are allowed *ad libitum* feed and water. Injections are most commonly administered IP, but SC, PO and IV dosing may be required on occasion. Dose volumes are generally 0.1 mL/10 grams body weight but may be up to 0.2 mL/10 grams of body weight for IP, IV, SC and PO routes. For further details visit [http://dtp.nci.nih.gov](http://dtp.nci.nih.gov/).

**Cell-Based Functional-VDR-Reporter Assay**

Analysis of VDR modulation by MT19c.

Effect of MT19c on VDR was examined by a cell-based functional VDR-reporter assay. Details of assay are available at (www.invitrogen.com; see SelectScreen® Cell-based Nuclear Receptor Profiling Services). This cellular reporter assay is based on enzyme beta-lactamase and a cell permeable FRET-paired fluorescent substrate that facilitates function-based assays of recombinant VDR-UAS-bla HEK 293T cells, which contain the ligand-binding domain (LBD) of the human Vitamin-D receptor (VDR) fused to the DNA binding domain of GAL4 stably integrated into the cell-line. UAS-bla HEK 293T cells stably express a beta-lactamase reporter gene under the transcriptional control of an upstream activator sequence (UAS). When an agonist binds to the LBD of the GAL4(DBD)-VDR(LBD) fusion protein, the protein binds to the UAS, resulting in expression of beta-lactamase. This approach allows for ratiometric data analysis of EC50/IC50 in both agonist (% activation) and antagonist (% inhibition) mode, using 10-point dose response curves.

Briefly, VDR-UAS-bla HEK 293T cells (10,000 cells/well) were grown in DMEM, 10% dialyzed FBS, 25mM HEPES, 0.1mM NEAA, 100U/mL/100μg/mL Pen/Strep) under selection (80μg/mL Zeocin, 80μg/mL Hygromycin B). Cells were dissociated and resuspended in assay media (DMEM phenol red free, 2% CD-treated FBS, 0.1mM NEAA, 1mM sodium pyruvate, 100U/mL/100μg/mL Pen/Strep) to a concentration of 625,000 cells/mL. Four (4) μL of a 10X serial dilution of Calcitriol (control agonist starting concentration, 1nM) or compounds were added to a 384-well TC-treated assay plate and 32μL of a cell suspension (10,000 cells) were added to each well. Assay media was added to all wells to bring the final assay volume to 40μL. The plate was incubated for 5h at 37ºC/5% CO2 in a humidified incubator. Cells were loaded with LiveBLAzer™-FRET B/G substrate (1μM final concentration of CCF4-AM) for 2h. Fluorescence was measured at 460nmem/530nmex. For antagonist effect determination, cells were stimulated with calcitriol (0.12 nanomoles) and co-treated with MT19c before the assay was carried out as described above.

**Determination of agonistic/antagonistis properties of MT19c using a PPAR-Ý–coactivator binding assay**

The assay has been described in detail previously ([[1]](#endnote-2)). Briefly, pET15b-PPARγ-LBD expression plasmid, encoding the PPARγ-LBD (amino acids 173-475) was a generous gift from Gabor J. Tigyi (University of Tennessee, Memphis). PPARγ-LBD was expressed in BL21 (DE3) (Invitrogen), purified by affinity chromatography, and stored at -80°C in buffer (50 mM Tris (pH 8.0), 25 mM KCl, 2 mM DTT, 10% glycerol, 0.01% NP-40). For the assay, MT19c were serially diluted in DMSO and 100 nl of each concentration was transferred into 20 µL protein buffer (20 mM TRIS (pH 7.5), 100 mM NaCl, 0.01 % NP-40, 2% DMSO, 10 nM DRIP2 (CNTKNHPMLMNLLKDNPAQD) labeled with Texas-Red maleimide, and 1 μM PPARγ-LBD) in the presence and absence of rosiglitazone (5 μM) in quadruplet using black 384 well plate (Costar, #3658). The samples were allowed to equilibrate for two hours. Binding was then measured using fluorescence polarization (excitation 595 nm, emission 615 nm) using a M1000 plate reader (Tecan). The experiments were evaluated using GraphPad Prism 5, and IC50 values were obtained by fitting the data to an equation (Sigmoidal dose-response-variable slope (four parameters). Values are given as the mean values of two independent experiments with a 95% confidence interval.

**Immunohistochemical analysis of tumors slides**

Immunohistochemical staining was performed on the vehicle or drug treated tumor specimens on paraffin-embedded slides (thickness 5μm). Tissue sections were deparaffinized and rehydrated with serial ethanol dilutions of 100, 95 and 70%. Heat-induced antigen retrieval was then performed using DAKO Antigen Retrieval Solution for 20 min. Tissue sections were blocked with Normal Goat Blocking Serum (Vector Laboratories) for 60 min at room temp. FITC-phosphoEGFR (R&D systems, dilution 1:100), FASN and phospho-acetyl-coA carboxylase primary antibodies (Cell Signaling Technologies, dilution 1:300) were added to completely cover tissue, incubated in a humidified chamber overnight at 4°C in a humidified chamber overnight at 4°C. Secondary antibodies (DyLight 594 goat anti-rabbit IgG, Jackson ImmunoResearch Laboratories, INC. and Alexa Fluor 594 goat anti-mouse IgG at 1:500, Invitrogen) were applied and incubated for 60 min for 1 hour at room temperature in the dark. Vectashield media with DAPI (Vector Laboratories) was used to mount cover-slips for further analysis. Sixteen bit images were acquired with a Nikon E800 microscope (Nikon Inc. Mellville NY) using a 40x PlanApo objective. A Spot II digital camera (Diagnostic Instruments, Sterling Heights MI) was used to acquire the images. The cameras built-in green filter was used to increase image contrast. Camera settings were based on the brightest slide. All subsequent images were acquired with the same settings. Image processing and analysis was performed using iVision (BioVision Technologies, version 10.4.11, Exton, PA.) image analysis software. Positive staining was defined through intensity thresholding and integrated optical density (IOD) was calculated by examining the thresholded area multiplied by the mean. All measurements were performed in pixels.

Confocal images were acquired with a Nikon C1si confocal (Nikon Inc. Mellville NY.) using diode lasers 402, 488 and 561. Serial optical sections were performed with EZ-C1 computer software (Nikon Inc. Mellville, NY). Z series sections were collected at 0.3µm with a 40x PlanApo lens and a scan zoom of 2. The gain settings were based on the brightest slide and kept constant between specimens. Deconvolution and projections were done in Elements (Nikon Inc. Mellville, NY) computer software.

**HPLC based quantification of cellular malonyl CoA content and Mass spectrometry based characterization.**

A Shimadzu HPLC system was used for sample analysis. The system consisted of SCL-10Avp controller, two LC-10ADvo pumps, and a SPD-10A detector. The HPLC was run in the binary mode with a Zorbax 300 Extend 300 C18, 3.5-µm column (2.1x50 mm, Agilent) and monitored at 254 nm. Solvent A consisted of 10mM NH4OAc, 5 mM dimethylbutyl amine, and solvent B was pure acetonitrile. The chromatographic conditions were as follow: The flow rate was set at 200 µL/min at room temperature, 2.5% to 12.5%B in 2 min; 12.5% to 25% B in 5 min, and then return to initial condition in 2 min.

**Statistical Analysis**

Data analysis was performed with STATA 9 (StataCorp, College Station, TX, USA) and SAS 9.1 (SAS Institute, Cary, NC, USA). Two-tailed p-values were presented, with p<0.05 considered statistically significant. Means were compared by Student's T-test with adjustment for unequal variances as appropriate. Tumor growth rates in the MT19c-treated mice and controls were analyzed by repeated measures linear regression. A first-order autoregressive covariance pattern was used to model the within-subject correlation. Treatment group and evaluation days were entered as factors in model along with their interaction term. Tumor growth was also examined by Kaplan-Meier analysis and the log-rank test. The outcome was growth of 10mm or more, and follow-up was censored at the end of evaluation (60 days) or at the time of euthanasia. The proportional hazards assumption was checked by graphical inspection.

Reference:

1. . Feau C, Arnold LA, Kosinski A, Zhu F, Connelly M and Guy RK. Novel flufenamic acid analogues as inhibitors of androgen receptor mediated transcription. ACS Chem Biol 2009;4:834-843. [↑](#endnote-ref-2)
